# An algorithm for calculating top-dimensional bounding chains

**DOI:** 10.7717/peerj-cs.153

**Published:** 2018-05-28

**Authors:** J. Frederico Carvalho, Mikael Vejdemo-Johansson, Danica Kragic, Florian T. Pokorny

**Affiliations:** 1 CAS/RPL, KTH, Royal Institute of Technology, Stockholm, Sweden; 2 Mathematics Department, City University of New York, College of Staten Island, New York, NY, United States of America

**Keywords:** Homology, Computational algebraic topology

## Abstract

We describe the Coefficient-Flow algorithm for calculating the bounding chain of an $(n-1)$-boundary on an $n$-manifold-like simplicial complex $S$. We prove its correctness and show that it has a computational time complexity of *O*(|*S*^(*n*−1)^|) (where *S*^(*n*−1)^ is the set of $(n-1)$-faces of $S$). We estimate the big- $O$ coefficient which depends on the dimension of $S$ and the implementation. We present an implementation, experimentally evaluate the complexity of our algorithm, and compare its performance with that of solving the underlying linear system.

## Introduction

Topological spaces are by and large characterized by the cycles in them (i.e., closed paths and their higher dimensional analogues) and the ways in which they can or cannot be deformed into each other. This idea had been recognized by Poincaré from the beginning of the study of topology. Consequently much of the study of topological spaces has been dedicated to understanding cycles, and these are the key features studied by the topological data analysis community (Edelsbrunner & Harer, 2010).

One key part of topological data analysis methods is to distinguish between different cycles; more precisely, to characterize different cycles according to their homology class. This can be done efficiently using cohomology (Pokorny, Hawasly & Ramamoorthy, 2016; Edelsbrunner, Letscher & Zomorodian, 2002). However, such methods only distinguish between non-homologous cycles, and do not quantify the difference between cycles. A possible way to quantify this difference is to solve the problem of finding the chain whose boundary is the union of the two cycles in question, as was proposed in Chambers & Vejdemo-Johansson (2015) by solving the underlying linear system, where the authors also take the question of optimality into account (with regards to the size of the resulting chain). This line of research ellaborates on Dey, Hirani & Krishnamoorthy (2011) where the authors considered the problem of finding an optimal chain in the same homology class as a given chain. More recently, in Rodríguez et al. (2017) the authors have taken a similar approach to ours using a combinatorial method to compute bounding chains of 1-cycles on 3-dimensional simplicial complexes.

In this paper, we explore the geometric properties of simplicial *n*-manifolds to provide an algorithm that is able to calculate a chain whose boundary is some prescribed (*n* − 1)-dimensional cycle, and we show that the proposed algorithm has a complexity which is linear in the number of (*n* − 1)-faces of the complex. This is substantially faster than calculating a solution to the linear system as considered in Chambers & Vejdemo-Johansson (2015), for which the complexity would be, at least, quadratic on the number of *n*-dimensional faces (Gloub & Van Loan, 1996).

### Background

In what follows we make extensive use of sequences. Therefore, for any *n* ∈ ℕ, we abbreviate *x*_0_, …, *x*_*n*_ to *x*_0:*n*_.

#### Simplicial complexes

Given a set of points *P*⊆ℝ^*n*^, we define a *k*-*dimensional simplex*, or *k*-simplex, on points of *P* as the ordered set [*p*_0:*k*_], where *p*_0:*k*_ ∈ *P* are *k* + 1 affinely independent points and are called the *vertices* of [*p*_0:*k*_]. We represent the simplex [*p*_0:*k*_] by the convex hull of the points *p*_0:*k*_, and we say that two simplices are the same if they have the same points and the ordering of the points differs only by an even permutation. If the ordering differs by an odd permutation we say they have opposite *orientations*.

Since a convex hull of a finite set of points is a bounded closed set, it carries the notion of a *boundary* ∂[*p*_0:*k*_] which is defined as:

\begin{eqnarray*}\partial [{p}_{0:k}]=[{p}_{1:k}]+(\sum _{i=1}^{k-1}(-1)^{i}[{p}_{0:i-1},{p}_{i+1:k}])+(-1)^{k}[{p}_{0:k-1}]. \end{eqnarray*}

The above sum can be interpreted as a “union with orientation”, and multiplying by 1 or −1 is the identity or a reversal of orientation, respectively. Note that if *p*_0:*k*_ are affinely independent, then the boundary of the convex hull does indeed correspond to the union of the convex hulls of all subsets of {*p*_0:*k*_} with k distinct points.

For example, the boundary of the simplex [*p*_0_, *p*_1_, *p*_2_] is given by:

\begin{eqnarray*}[{p}_{0},{p}_{1}]-[{p}_{0},{p}_{2}]+[{p}_{1},{p}_{2}] \end{eqnarray*}

Applying the only possible orientation-reversing permutation to [*p*_0_, *p*_2_] gives [*p*_0_, *p*_1_] + [*p*_1_, *p*_2_] + [*p*_2_, *p*_0_]. This corresponds to the union of the edges that form the boundary of the triangle [*p*_0_, *p*_1_, *p*_2_] oriented in such a way as to form a *closed path*.

Definition 1. *A set of points P*⊆ℝ^*n*^* and a set of simplices T* = {*σ*_0:*N*_}* defines a*geometric simplicial complex* S* = (*P*, *T*)* if any finite subset of a simplex in T is also in T, and given any two simplices* [*p*_0:*k*_], [*q*_0:*k*′_] ∈ *T, the intersection of the convex hulls* [*p*_0:*k*_]∩[*q*_0:*k*′_]* is the convex hull of* {*p*_0:*k*_}∩{*q*_0:*k*′_}* and is also a simplex in T*.

* For any d we define the d-skeleton of T by T*^*d*^ = {*σ* ∈ *T*∣dim*σ* ⩽ *d*}* and the d-th*level* as T*^(*d*)^ = {*σ* ∈ *T*∣dim*σ* = *d*}*.*

Given two simplices *σ*, *τ* we write *τ*◃*σ* if *τ* ⊂ *σ* and dim*τ* = dim*σ* − 1 which can be read as “*τ* is a top-dimensional face of *σ*”. Note that ◃ is not transitive, and therefore it is not a preorder. Its transitive closure, *τ* < *σ* however, defines a preorder. We can thus read *τ* < *σ* as “*τ* is contained in the boundary of *σ*” or simply “*τ* is a *face* of *σ*”.

We say that a simplicial complex *S* = (*P*, *T*) has *dimension d* if *d* is the largest integer such that *T*^(*d*)^ ≠ ∅.

Definition 2. *A d-dimensional simplicial complex S* = (*P*, *T*)* is called a d-*manifold-like* simplicial complex if*

• *for every τ* ∈ *T*^*d*−1^ *there exists some σ* ∈ *T*^(*d*)^ *such that σ* > *τ*, *and*
• *if* dim*τ* = (*d* − 1) *then there are at most two σ* ∈ *T*^(*d*)^ *satisfying σ* > *τ*.

Note that a triangulation of a *d*-manifold is a manifold-like simplicial complex, however the definition also includes other spaces like triangulations of manifolds with boundary and the pinched torus.

#### Algebraic description

We will focus on finite geometric simplicial complexes *S* = (*P*, *T*) (where |*P*|, |*T*| < ∞). Since such an *S* has a finite number of simplices, we can define for each level 0 ⩽ *k* ⩽ dim(*S*) an injective function *ι*_*k*_:*T*^(*k*)^ → ℕ such that *ι*_*k*_(*T*^(*k*)^) = {1, …, |*T*^(*k*)^|}; we call *ι* an *enumeration* of *T*^(*k*)^. From this we define the *chain complex* associated with *S*.

Definition 3. *Given a simplicial complex S* = (*P*, *T*)* the* chain complex* associated with S is defined as the pair* ${\{({C}_{k}(S),{d}_{k})\}}_{k=0}^{+\infty }$* where the C*_*k*_(*S*)* are vector spaces defined as C*_*k*_(*S*) = ℝ^|*T*^(*k*)^|^* and the d*_*k*_* are linear maps d*_*k*_:*C*_*k*_(*S*) → *C*_*k*−1_(*S*)* defined on basis elements as*

\begin{eqnarray*}{d}_{k}({e}_{i})=\sum _{\tau \in \partial ({\iota }_{k}^{-1}(i))}o(i,\tau ){e}_{{\iota }_{k-1}(\tau )} \end{eqnarray*}

*where o*(*i*, *τ*)* is the orientation of τ induced by the boundary of* $\sigma ={\iota }_{k}^{-1}(i)$*.*

It can be shown that *d*_*k*_∘*d*_*k*+1_ = 0, which allows us to define, for each *k*, the *k*-th homology group of *S* as

\begin{eqnarray*}{H}_{k}(S)=\ker \nolimits ({d}_{k})/\text{im}({d}_{k+1}). \end{eqnarray*}

By a slight abuse of notation, for a simplicial complex *S* = (*P*, *T*) and a *k*-chain *c*, we write *c*_*σ*_ for the coefficient corresponding to *σ*, *e*_*σ*_ for the corresponding basis element and *d* for the appropriate boundary map whenever these are clear from their contexts.

We call the elements *p* ∈ *C*_*k*_(*S*) such that *dp* = 0, *k*-*cycles*. Two *k*-cycles *p*, *p*′ are said to be *homologous* if there exists a chain *c* ∈ *C*_*k*+1_(*S*) such that *dc* = *p* − *p*′, so that *p* − *p*′ is called a *k*-*boundary*. This defines *k*-homology as the group of *k*-cycles quotiented by the homology relation.

### Problem description and contribution

We are interested in the bounding chain problem, that is, given a cycle *p*, we want to decide whether or not *p* is a boundary, and in case it is, provide a witness in the form of a chain *c* such that ∂*c* = *p*; we call *c* a *bounding chain* of *p*. To achieve this, we further specialize the problem to

\begin{eqnarray*}\text{solve:}\partial c=p \end{eqnarray*}

(1) \begin{eqnarray*}\text{subject to:}{c}_{\sigma }=v,\end{eqnarray*}

where *p* is a specified (*n* − 1)-boundary in an *n*-manifold-like simplicial complex *S*, *σ* is an *n*-simplex, and *v* is a pre-specified real number. In general, the equation ∂*c* = *p* has more than one solution *c*, therefore by adding the constraint *c*_*σ*_ = *v* we are able to make this solution unique.

The Coefficient-Flow algorithm that we present solves this restricted form of the bounding chain problem (by providing one such bounding chain if it exists) and has computational time complexity of *O*(|*S*^(*n*−1)^|). Furthermore, we show how the parameters *σ* and *v* can be done away with in cases where the chain is unique, and we discuss how this algorithm can be used to find a minimal bounding chain.

### Related work

In Chambers & Wang (2013) the authors address the problem of computing the area of a homotopy between two paths on 2-dimensional manifolds, which can be seen as a generalization of the same problem, for 2-dimensional meshes via the Hurewicz map (Hatcher, 2002). In Chambers & Vejdemo-Johansson (2015) the authors provide a method for calculating the minimum area bounding chain of a 1-cycle on a 2d mesh, that is the solution to the problem

(2) \begin{eqnarray*}\arg \nolimits \,{\min \,}_{c}\text{area}(c)=p, \text{where}\partial c=p\end{eqnarray*}

and *p* is a 1-chain on a given simplicial complex. This is done by using optimization methods for solving the associated linear system. These methods however have time complexity lower-bounded by matrix multiplication time which is in Ω(min(*n*, *m*)^2^) where *n*, *m* are the number of rows and columns of the boundary matrix^1^ 1Which corresponds to the number of (*k* − 1)- and *k*-faces of the complex, respectively. (Davie & Stothers, 2013). This complexity quickly becomes prohibitive when we handle large complexes, such as one might find when dealing with meshes constructed from large pointclouds.

More recently, in Rodríguez et al. (2017) the authors proposed a method for computing bounding chains of 1-cycles in 3-dimensional complexes, using a spanning tree of the dual graph of the complex.

In Dey, Hirani & Krishnamoorthy (2011) the authors address the related problem of efficiently computing an optimal cycle *p*′ which is homologous to a given cycle *p* (with ℤ coefficients). This is a significant result given that in Chambers, Erickson & Nayyeri (2009) the authors proved that this cannot be done efficiently (i.e., in polynomial time) for 1-cycles using ℤ_2_ coefficients, a result that was extended in Chen & Freedman (2011) to cycles of any dimension.

## Methodology

For any simplicial complex *S*, and any pair of simplices *σ*, *τ* ∈ *S* such that, *τ* ◃ *σ*, we define the index of *τ* with respect to *σ* as 〈*τ*, ∂*σ*〉 = 〈*e*_*τ*_, *de*_*σ*_〉 (Forman, 1998). Note that the index corresponds to the orientation induced by the boundary of *σ* on *τ* and can be computed in *O*(*d*) time by the following algorithm:


 
___________________________________________________________________________ 
Index(σ,τ): 
     param: σ - k-simplex represented as a sorted list of indices of points. 
     param: τ - (k − 1)-face of σ represented as a sorted list of indices. 
1: for each   i ← 0...dimτ : 
2:      if   τi ⁄= σi: 
3:         orientation ← (−1)i 
4:         break loop 
5: return orientation 
___________________________________________________________________________    


By inspecting the main loop we can see that Index (*σ*, *τ*) returns (−1)^*i*^ where *i* is the index of the first element at which *σ* and *τ* differ. We assume *τ* is a top dimensional face of *σ*, so if *σ* = [*s*_0:*d*_], then by definition *τ* = [*s*_0:(*i*−1)_, *s*_(*i*+1):*d*_] for some *i*, and so the coefficient of *τ* in the boundary of *σ* is (−1)^*i*^ as per the definition of the boundary operator. This is also the index of the first element at which *τ* and *σ* differ, since they are represented as sorted lists of indices.

The following is an intuitive result, that will be the basis for the Coefficient-Flow algorithm that we will present in the sequel.

Proposition 1. * Let S be a manifold-like simplicial complex, let c be an n-chain on S and p its boundary. Then for any pair of n-simplices σ* ≠ *σ*′* with* ∂*σ*∩∂*σ*′ = {*τ*}* we have:*

\begin{eqnarray*}{c}_{\sigma }=\langle \tau ,\partial \sigma \rangle ({p}_{\tau }-\langle \tau ,\partial {\sigma }^{{^{\prime}}}\rangle {c}_{{\sigma }^{{^{\prime}}}}). \end{eqnarray*}

**

*Proof.* If we expand the equation ∂*c* = *p*, we get *p*_*τ*_ = ∑_*τ*◃*ω*_〈*e*_*τ*_, *d*(*c*_*ω*_*e*_*ω*_)〉, recall that by definition *d*(*c*_*ω*_*e*_*ω*_) = ∑_*ν*◃*ω*_〈*ν*, ∂*ω*〉*c*_*ω*_*e*_*ν*_; and so we get *p*_*τ*_ = ∑_*τ*◃*ω*_〈*τ*, ∂*ω*〉*c*_*ω*_*e*_*τ*_.

Now since *S* is a manifold-like simplicial complex and *τ* = ∂*σ*∩∂*σ*′, then *σ*, *σ*′ are the *only* cofaces of *τ*, and hence we have:

\begin{eqnarray*}{p}_{\tau }=\langle \tau ,\partial \sigma \rangle {c}_{\sigma }+\langle \tau ,\partial {\sigma }^{{^{\prime}}}\rangle {c}_{{\sigma }^{{^{\prime}}}} \end{eqnarray*}

which can be reorganized to ${c}_{\sigma }= \frac{{p}_{\tau }-\langle \tau ,\partial {\sigma }^{{^{\prime}}}\rangle {c}_{{\sigma }^{{^{\prime}}}}}{\langle \tau ,\partial \sigma \rangle } $. Finally, since the index 〈*τ*, ∂*σ*〉 is either 1 or −1, we can rewrite this equation as:

\begin{eqnarray*}{c}_{\sigma }=\langle \tau ,\partial \sigma \rangle ({p}_{\tau }-\langle \tau ,\partial {\sigma }^{{^{\prime}}}\rangle {c}_{{\sigma }^{{^{\prime}}}}) \end{eqnarray*}

Next, we present an algorithm to calculate a bounding chain for a (*n* − 1)-cycle in an *n*-manifold-like simplicial complex. The algorithm proceeds by checking every top-dimensional face *σ*, and calculating the value of the chain on adjacent top-dimensional faces, using Proposition 1.

In order to prove that the Coefficient-Flow algorithm solves problem (1), will use the fact that we can see the algorithm as a traversal of the *dual graph*.

Definition 4. *Given an n-dimensional simplicial complex S, recall that the*dual graph* is a graph G*(*S*) = (*V*, *E*)* with set of vertices V* = *S*^(*n*)^* and* (*σ*, *σ*′) ∈ *E if* dim(*σ*∩*σ*′) = *n* − 1*.*

 
________________________________________________________________________________________________________________________________________________ 
 
Coefficient-Flow(p,σ0,v0): 
     param: p — an n − 1 boundary of the simplicial complex S 
    param: σ0 — an n–simplex from where the calculation will start 
     param: v0 — the value to assign the bounding chain at sigma 
     return: c — a bounding chain of p satisfying cσ0  = v0 (if it exists) 
6:   initialize c and mark every n- and (n − 1)-cell of S as not seen. 
7:   initialize an empty queue Q 
8:   let τ0 ∈ ∂σ0 
9:   enqueue (σ0,τ0,v0) into Q. 
10:   while   Q is non-empty: 
11:       (σ,τ,v) ← pop first element from Q 
12:       if   σ has been marked seen: 
13:            if   v ⁄= cσ: the problem has no solution 
14:       else: 
15:            if   τ has been marked seen: skip 
16:            cσ ← v 
17:            mark τ and σ as seen 
18:            for each   τ′ ∈ ∂σ: 
19:                if   σ is the only coface of τ′: 
20:                    mark τ′ as seen 
21:                    if   pτ′ ⁄= 〈∂σ,τ′〉v : the problem has no solution 
22:                else: 
23:                    if   τ′ has not been marked as seen: 
24:                        σ′ ← other coface of τ′ 
25:                        v′ ←〈∂σ′,τ〉(pτ −〈∂σ,τ〉v) 
26:                        enqueue (σ′,τ′,v′) into Q 
27:   return c 
__________________________________________________________________________________________________________________________________

Proposition 2. * If S is a manifold-like simplicial complex, where G*(*S*)* is connected, and p is an* (*n* − 1)*-boundary, then* Coefficient- Flow (*p*, *σ*, *v*)* returns a bounding chain c of p satisfying c*_*σ*_ = *v, if such a boundary exists. Furthermore, the main loop (05–21) is executed at most O*(|*S*^(*n*−1)^|)* times.*

*Proof.* We start by proving the bound on the number of executions of the main loop. This is guaranteed by the fact that the loop is executed while the queue is non-empty, and a triple (*σ*, *τ*, *v*) can only be inserted in line (21) if *τ* has not been marked as seen. Furthermore, since *τ* has at most two cofaces, say, *σ*, *σ*′, we can only enqueue *τ* if we are analyzing *σ* or *σ*′ and so for each *τ*, at most two elements are placed in the queue, and hence the main loop gets executed at most 2|*S*^(*n*−1)^| times.

To prove correctness of the algorithm, we have to prove that it outputs an error if and only if the problem has no solution, and otherwise it outputs a bounding chain of *p* with *c*_*σ*_0__ = *v*.

First, we note that if a face *σ* is marked as seen, the value of *c*_*σ*_ can never be reassigned. This is because the program branches on whether or not *σ* has been marked as seen, and *c*_*σ*_ can only be assigned on line (11) which is bypassed if *σ* has been previously marked as seen. From this fact we conclude that *c*_*σ*_0__ = *v*_0_ as it is assigned on line (11) and *σ*_0_ is marked as seen in the first iteration of the main loop.

Second, note that there is an edge between two *n*-faces in the dual graph if and only if they share an (*n* − 1)-face. This implies that as we execute the algorithm, analyze a new *n*-face *σ* and successfully add the other cofaces of elements of the boundary of *σ*, we add the vertices neighboring *σ* in the dual graph. Since the dual graph is connected all of the nodes in the graph are eventually added, and hence all of the *n*-faces are analyzed.

Third, we note that for any pair (*τ*, *σ*) with dim*σ* = *n* and *τ* ◃ *σ*, either *σ* is the only coface of *τ*, or *τ* has another coface, *σ*′. In the first case, if *p*_*τ*_ ≠ *c*_*σ*_〈*τ*, ∂*σ*〉 an error is detected on line (16). In the second case, assuming that the triple (*σ*, *τ*, *v*) is enqueued before (*σ*′, *τ*, *v*′) we have *v*′ = 〈∂*σ*′, *τ*〉(*p*_*τ*_ − 〈∂, *σ*〉*v*) as is assigned in line (20) then

\begin{eqnarray*}(dc)_{\tau }=\langle \partial \sigma ,\tau \rangle v+\langle \partial {\sigma }^{{^{\prime}}},\tau \rangle {v}^{{^{\prime}}}\nonumber\\\displaystyle =\langle \partial \sigma ,\tau \rangle v+\langle \partial {\sigma }^{{^{\prime}}},\tau \rangle (\langle \partial {\sigma }^{{^{\prime}}},\tau \rangle ({p}_{\tau }-\langle \partial ,\sigma \rangle v))\nonumber\\\displaystyle =\langle \partial \sigma ,\tau \rangle v+{p}_{\tau }-\langle \partial \sigma ,\tau \rangle v={p}_{\tau }. \end{eqnarray*}

Finally, since upon the successful return of the algorithm, this equation must be satisfied by every pair *τ*◃*σ*, it must be the case that *dc* = *p*. If this is not the case, then there will be an error in line (08) and the algorithm will abort.□

Note that the connectivity condition can be removed if we instead require a value for one cell in each connected component of the graph *G*(*S*), and throw an error in case there is an (*n* − 1)-simplex *τ* with no cofaces, such that *p*_*τ*_ ≠ 0. Furthermore, the algorithm can be easily parallelized using a thread pool that iteratively processes elements from the queue.

Finally, in the case where it is known that *S* has an (*n* − 1)-face *τ* with a single coface, we do not need to specify *σ* or *v* in Coefficient-Flow, and instead use the fact that we know the relationship between the coefficient *p*_*τ*_ and that of its coface in a bounding chain *c* of *p*, i.e., *p*_*τ*_ = 〈∂*σ*, *τ*〉*c*_*σ*_. This proves Corollary 1.

 
____________________________________________________________________________________________________________________________________________________ 
 
Bounding-Chain(p): 
     param: p — an n − 1 boundary of the simplicial complex S 
    return: c — a bounding chain of p 
28:   for each   τ ∈ Sn−1: 
29:       if   τ has a single coface: 
30:            σ ← the coface of τ 
31:            v ←〈∂σ,τ〉pτ 
32:            break loop 
33:   c ← Coefficient-Flow(p,σ,v) 
34:   return c 
_______________________________________________________________________________________________________________________________________

Corollary 1. * If S is a connected n-manifold-like simplicial complex with a connected dual graph, and has an* (*n* − 1)*-face with a single coface, then given an* (*n* − 1)*-cycle p on S,* Bounding-Chain (p)* returns a bounding chain of p if one exists.*

### Implementation details

We will now discuss some of the choices made in our implementation of the Coefficient-Flow algorithm (https://www.github.com/crvs/coeff-flow). Before we can address the problem of considering chains on a simplicial complex we first need to have a model of a simplicial complex. For this, we decided to use a simplex tree model (Boissonnat & Maria, 2014) provided by the GUDHI library (The GUDHI Project, 2015) as it provides a compact representation of a simplicial complex (the number of nodes in the tree is in bijection with the number of simplices) which allows us to quickly get the enumeration of a given simplex *σ*. Indeed, the complexity of calculating *ι*_*k*_(*σ*) is in *O*(dim*σ*).

It is important to compute the enumeration quickly because in our implementation of Coefficient-Flow we use arrays of booleans to keep track of which faces of the simplicial complex have been seen before, as well as numerical arrays to store the coefficients of the cycle and its bounding chain, which need to be consulted at every iteration of the loop.

However, finding the cofaces of the simplicial complex is not as easy in a simplex tree, since, if *σ* = [*p*_*i*_0_:*i*_*k*__], this would require to search every child of the root node of the tree that has an index smaller than *i*_0_, followed by every child of the node associated with *p*_*i*_0__ and so on, which in the worst case scenario is in *O*(dim*σ*|*S*^(0)^|). Thus we need to adopt a different method. For this, we keep a Hasse diagram of the face relation which comprises a directed graph whose vertices are nodes in the simplex tree and has edges from each face to its codimension-1 cofaces (see for instance Di Battista & Tamassia (1988)) for more details of this data-structure). This allows us to find the codimension-1 cofaces of a simplex of *S* in *O*(1) with a space overhead in *O*(|*S*|).

With these elements in place, we can analyze the complexity of our implementation of the Coefficient-Flow algorithm in full:

Lemma 1. *The Coefficient-Flow algorithm using a simplex tree and a Hasse diagram has computational (time) complexity O*(*d*^2^|*S*^(*d*−1)^|)* where d* = dim*S.*

*Proof.* In Proposition 2 we saw that the Coefficient-Flow algorithm executes the main loop at most *O*(|*S*^(*d*)^|) times, so we only need to measure the complexity of the main loop, in order to obtain its time complexity. This can be done by checking which are the costly steps:

• In lines (07) and (10) checking whether a face has been marked as seen requires first computing *ι*_*k*_(*τ*) which, as we stated above, has time complexity *O*(*k*), with *k* ≤ *d*.
• In line (13), computing the faces of a simplex *σ* requires *O*(dim*σ*^2^) steps, and yields a list of size dim*σ*, hence the inner loop (13–21) is executed dim*σ* times, where dim*σ* = *d*, for each element placed in the queue.
• The loop (13–21) requires once again, computing *ι*(*τ*′), and 〈∂*σ*, *τ*〉, each of these operations, as we explained before, carries a time complexity *O*(*d*).
• All other operations have complexity *O*(1).

Composing these elements, the total complexity of one iteration of the main loop is *O*(max{*d*, *d*^2^, *d*⋅*d*}) = *O*(*d*^2^), which yields a final complexity for the proposed implementation, of *O*(*d*^2^|*S*^(*d*−1)^|).□

### Example runs and tests

**Figure 1:**
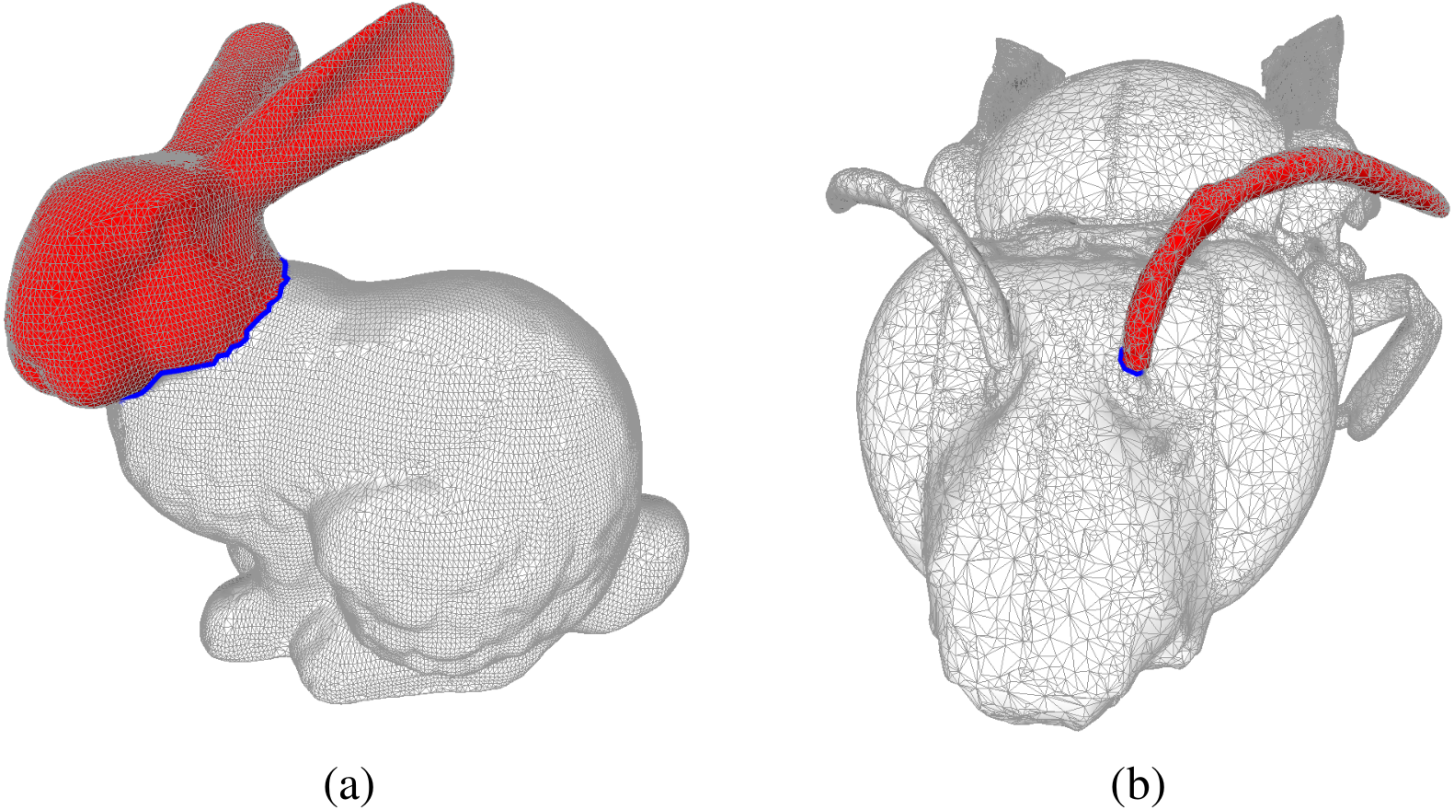
Examples of bounding chains: the edges in blue form cycles in the mesh, and the faces in red form the corresponding bounding chain as computed by the Coefficient-Flow algorithm. In (A) we depict the mesh of the Stanford Bunny The Stanford 3D scanning repository (1994) and in (B) we show the mesh of a Bee Smithsonian (2013). In both these meshes we depict examples of bounding chains, where the edges in blue form cycles, and the faces in red form the corresponding bounding chain as computed by the Coefficient-Flow algorithm. In both cases the depicted bounding chains correspond to the optimal bounding chains (w.r.t. the number of faces), these can by obtained by choosing *σ*_0_ and *v*_0_ so as to yield the desired chain. In this case, since the two complexes are topological spheres and the cycles are simple cycles (meaning they are connected and do not self-intersect), there are only two possible bounding chains that do not include all the faces of the complex, which can be obtained by running the algorithm three times, choosing *σ*_0_ arbitrarily, and setting *v*_0_ to be 0, *n* or −*n* where *n* = max_*τ*∈*S*^(1)^_|*p*_*τ*_|. In the case of non-simple cycles, more alternatives would exist.

In Fig. 1 we provide an example of the output of the Coefficient-Flow algorithm for the mesh of the Stanford bunny (The Stanford 3D scanning repository, 1994) and the *eulaema meriana* bee model from the Smithsonian 3D model library (Smithsonian, 2013).

For comparison, we performed the same experiments using the Eigen linear algebra library (Eigen, 2017) to solve the underlying linear system,^2^ 2We use the least squares conjugate gradient descent method to solve the system.and summarized the results in Table 1. This allowed us to see that even though both approaches remain feasible with relatively large meshes, solving the linear system consistently underperforms using the Coefficient-Flow algorithm.

**Table 1:** Timing for computation of bounding chains using Coefficient-Flow, and using Eigen in several meshes. “Bunny” and “Bee (Sample)/Bee (Full)” refer to the meshes in Figs. 1A and 1B, respectively. The mesh “Bee (Full)” is the one obtained from Smithsonian (2013), whereas the one labeled “Bee (Sample)” is a sub-sampled version of it.

Mesh	Faces	Edges	Vertices	Eigen	Coefficient-Flow
Bunny	69 663	104 496	34 834	2.073 (s)	**0.48911 (s)**
Bee (Sample)	499 932	749 898	249 926	116.553 (s)	**3.04668 (s)**
Bee (Full)	999 864	1 499 796	499 892	595.023 (s)	**7.15014 (s)**

**Figure 2:**
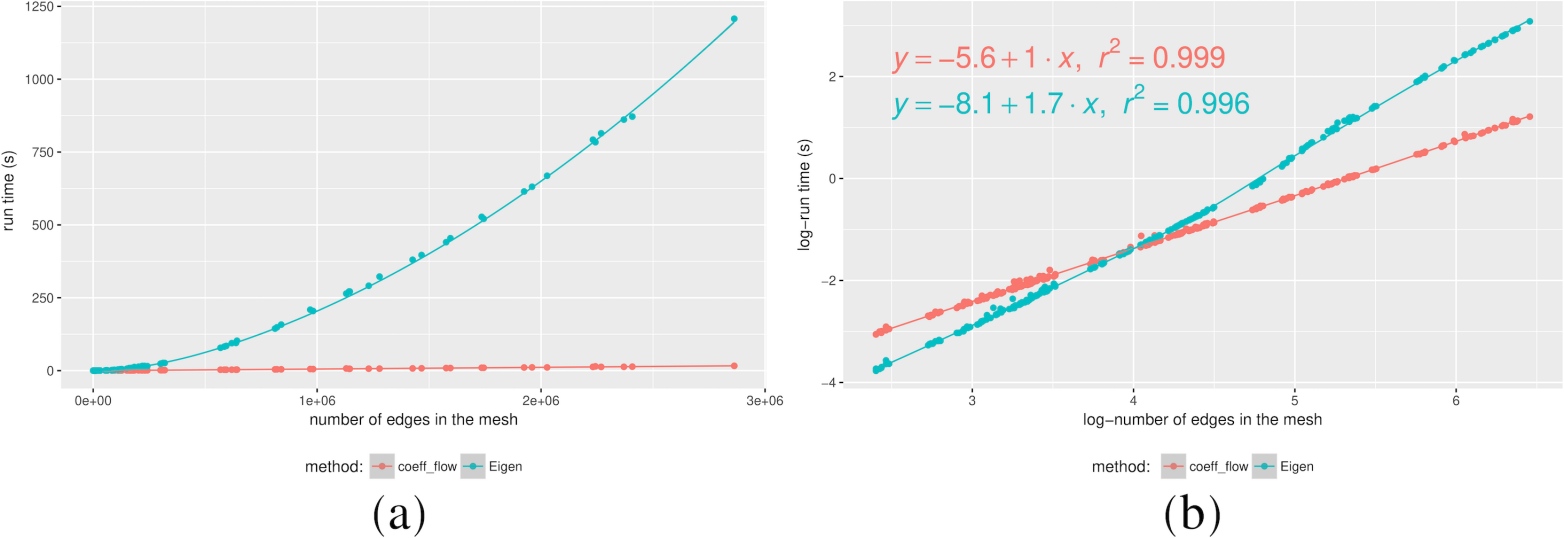
Running times for a calculating the bounding chain of a cycle as a function of the number of edges (A), and Log–log plot of the same data (B).

Even though Coefficient-Flow is expected to outperform a linear system solver (an exact solution to a linear system has Ω(*n*^2^) time complexity), we wanted to test it against an approximate sparse system solver. Such solvers (e.g., conjugate gradient descent (Gloub & Van Loan, 1996)) rely on iterative matrix products, which in the case of boundary matrices of dimension *d* can be performed in *O*((*d* + 1)*n*) where *n* is the number of *d*-dimensional simplices, placing the complexity of the method in Ω((*d* + 1)*n*). In order to observe the difference in complexity class we performed randomized tests on both implementations. In this scenario we made a mesh on a unit square from a random sample. By varying the number of points sampled from the square, we effectively varied the resolution of the mesh. Finally, at each resolution level we snapped a cycle onto the mesh, and computed its bounding chain using both Coefficient-Flow and by solving the sparse linear system as before. We plotted the timings in Fig. 2 from where we can experimentally observe the difference in the complexity class between our algorithm and the solution to the sparse linear system.

Furthermore by analyzing the Log-Log plot in Fig. 2B, we can confirm our complexity estimates by analyzing the slope of the lines where the samples are distributed, i.e., solving the sparse linear system is approximately *O*(*n*^1.7^) complexity,^3^ 3Since boundary matrices are naturally sparse, and we are computing an approximate solution, the complexity can be improved beyond the aforementioned Ω(*n*^2^).whereas Coefficient-Flow displays linear complexity.

## Conclusion and future work

While the problem of finding a bounding chain for a given cycle in a simplicial complex remains a challenging one for large complexes, we showed that this problem can be solved efficiently for codimension-1 boundaries. We implemented and tested our algorithm and have provided complexity bounds for its run-time.

However, this leaves open the question of finding bounding chains for boundaries of higher codimension, for which solving a large sparse linear system is still, to the best of our knowledge, the only feasible approach, save for codimension 2 cycles in dimension 3 (Rodríguez et al., 2017). In the future we would like to generalize our algorithm to be able to work with cobounding cochains (i.e., in cohomology), as well as considering the optimality question (i.e., finding the minimal bounding chain w.r.t. some cost function).

##  Supplemental Information

10.7717/peerj-cs.153/supp-1 Supplemental Information 1 Data used to produce the (timing) results presented in the text Files with data used to produce Table 1 and the graphs in Figure 2. All timings were obtained by running the tests available in the code repository https://www.github.com/crvs/coeff-flow.

## Additional Information and Declarations

### Data Availability

The following information was supplied regarding data availability: Github: https://www.github.com/crvs/coeff-flow.
